# Electrodialysis Can Lower the Environmental Impact of Hemodialysis

**DOI:** 10.3390/membranes12010045

**Published:** 2021-12-29

**Authors:** Ahmed Abarkan, Nabil Grimi, Hubert Métayer, Tarik Sqalli Houssaïni, Cécile Legallais

**Affiliations:** 1 Biomechanics & Bioengineering Laboratory, CNRS, Université de Technologie de Compiègne, 60203 Compiegne, France; ahmed.abarkan@utc.fr; 2 Laboratory of Epidemiology and Research in Health Sciences (ERESS), Faculty of Medicine and Pharmacy, Sidi Mohammed Ben Abdellah University, Fez 30050, Morocco; tarik.sqalli@usmba.ac.ma; 3 Transformations Intégrées de la Matière Renouvelable (TIMR), Université de Technologie de Compiègne, ESCOM, 60203 Compiegne, France; nabil.grimi@utc.fr; 4 Hemodialysis Service, Polyclinique Saint-Côme, 7 Rue Jean-Jacques Bernard, 60204 Compiegne, France; hubert.metayer@stcome.com; 5 La Dialoise Self-Dialysis Center, 5 Rue Jean-Jacques Bernard, 60200 Compiegne, France; 6 Department of Nephrology, University Hospital Hassan II, Fez 30050, Morocco

**Keywords:** hemodialysis, electrodialysis, demineralization rate, specific energy consumption, reverse osmosis rejects

## Abstract

The hemodialysis technique, used worldwide for patients with chronic kidney disease, is considered as a treatment with a high economic and ecological impact, especially for water consumption. Getting ultrapure water for the preparation of the dialysate to clean patient’s blood from toxins leads to high volumes of salt-enriched water that directly goes to sewage. The aim of this work is to propose operating conditions for electrodialysis to allow the reuse of reverse osmosis (RO) rejects. We first performed a parametric study to evaluate the influence of different parameters, such as flow rates, initial concentration, and applied voltage on the demineralization rate (DR) and specific energy consumption (SPC) with a NaCl model solution. The optimal conditions for desalination (i.e., a potential of 12 V, and flow rate of 20 L·h^−1^) were then successfully applied to real samples collected from a dialysis center with total dissolved salts concentration of about 1.4 g/L (conductivity of 2.0 mS·cm^−1^). We demonstrated that the choice of adequate conductivity targets allowed meeting the physico-chemical requirements to obtain water re-usable for either rehabilitation swimming pool, manual or machine washing of instruments before sterilization or irrigation. Saving this water could contribute in the reduction of the environmental impact of hemodialysis.

## 1. Introduction

**Figure 1:**
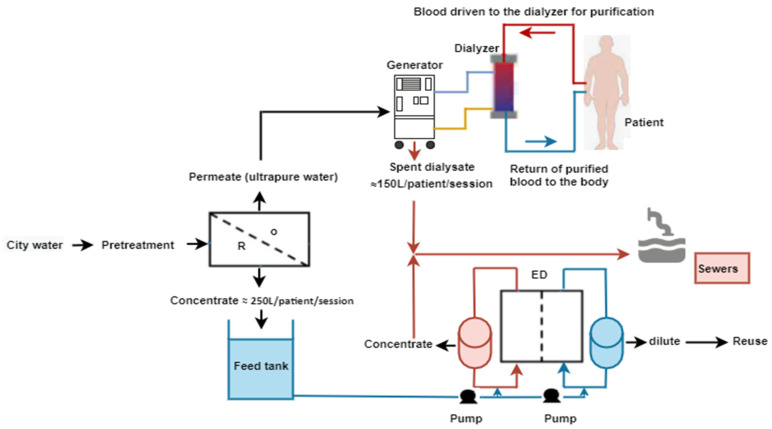
Schematic representation of the hydraulic circuits for the achievement of an hemodialysis session. The hemodialysis machine controls both the extracorporeal circulation and the exchanges through the membrane dialyzer with the dialysate that is prepared on-line by the dilution of concentrate solutions (ionic and bicarbonate) with the ultrapure water prepared in the treatment loop.

Water is an essential raw material for life on earth. Its scarcity is a major problem in many countries, including some in Europe, with climate changes. Hemodialysis (HD), i.e., membrane-based artificial kidney in an extracorporeal circulation, is a treatment with a high economic and ecological impact, especially regarding water consumption. Indeed, the preparation of the dialysate, which collects the uremic toxins after mass transfer from the blood in the dialyzer, requires a high-quality water (Figure 1). Tap water flows in a pre-treatment loop based on sand filters, activated carbon, ion-exchangers, and reverse osmosis (RO) [1]. The first RO stage rejects a mildly salted water, which is usually returned to sewage. The volume can reach up to 250 L per dialysis session.

In 2018, there were an estimated 3,362,000 people on dialysis worldwide, with 2,993,000 (89%) on hemodialysis [2,3]. The global dialyzed population grows every year and is expected to reach nearly 5 million people by 2025 [4]. At that time, and knowing that each patient undergoes three HD session per week, the volume of rejected water for RO loop per year will reach more than 200 million of m^3^.

Physicochemical data from the literature show that hemodialysis RO concentrates are mildly concentrated in sodium, nitrate, sulfate, and chloride ions [5]. Their partial demineralization/desalination is thus mandatory before their reuse in irrigation, cleaning, washing of vehicles, feeding of boilers, sterilization, or rehabilitation pool. Indeed, each reuse alternative should meet a more or less well defined water quality, according to national or international guidelines or standards, such as the French association of sterilization (AFS) for sterilization, World Health Organization, Food and Agriculture Organization WHO/FAO for irrigation, and the Regional Health Agency (ARS) for the rehabilitation pool and standards of toilet flushing [6,7].

Desalination techniques are used to prepare adequate-quality water from salted water. They mainly rely on two different physical means [8]. On the one hand, thermal technologies involving a phase change (distillation) [9] are rarely used to desalinate brackish water, since they are not cost effective and demand high energy for this application [10]. On the other hand, membrane-based technologies such as reverse osmosis (RO) are already well established. Electrodialysis (ED) is less energy intensive [11] and has recently attracted a lot of attention [12,13]. They have shown great potential to replace or complement conventional methods such as distillation and evaporation, as they are economical and safe [14]. Nanofiltration consumes less energy, and offers the great advantage of lower operating costs than RO and ED [15]; but a major drawback is that it results in lower monovalent salt rejections [16]. Therefore, NF is not an alternative for desalination of RO concentrates.

Electrodialysis (ED) is a membrane-based separation process in which ionized species such as salts and acids are transferred across an ion-exchange membrane from one solution to another by imposition of an electrical potential [14]. ED has been used for more than 60 years for the production of water from brackish water and seawater, municipal and industrial wastewater treatment, salt production from seawater and its application in chemical processes, food and pharmaceutical industries [17,18]. The operation of ED does not require chemicals and is more environmentally friendly [19]. It competes with reverse osmosis in that it allows for control of the salt concentration in the product water and changes the salt composition to meet the specific water quality requirements of each use [20,21].

Unlike RO, which requires mixing and post-treatment and has a lower recovery rate, ED has higher water recovery rates [22], easy operation, long membrane life [23,24], and selective separation of monovalent ions (such as Na^+^, Cl^−^, and NO_3_^−^) from multivalent ions (e.g., Ca^2+^, Mg^2+^, SO_4_^2−^) [17,23,25]. It is more resistant to fouling, which can also reduce the quality of the treated effluent due to the reduced selectivity of the membrane [23]. In this specific application, fouling is expected to be limited since both the drinking water origin and the pre-treatment phase result in a low content of organic matter and/or microorganisms in the dialysis RO wastewater [26].

Despite these promises, ED has not been proposed yet for implementation in the water treatment loop of hemodialysis centers, whereas environmental consideration is increasing in health systems. One of the hurdles is cost, which might be significant if the process is not correctly dimensioned. The aim of this work is therefore to study the efficiency of the ED process on the desalination of dialysis RO effluents, in order to prove their compatibility with on-site reuse. This efficiency is evaluated by the demineralization rate (DR), specific power consumption (SPC), and the ionic flux (J). First, experiments were conducted on NaCl model solutions to define optimal conditions using a laboratory-scale electrodialysis cell. The process was then applied to the treatment of RO water samples collected in a hemodialysis center, using the same conditions. The evaluation of the process to produce different types of reusable water are finally discussed, according to the standards or guidelines.

## 2. Materials and Methods

### 2.1. Real Dialysis RO Rejects Samples

The dialysis RO rejects studied were taken from the polyclinic Saint Come and La Dialoise centers, Compiègne, France. The physico-chemical characteristics of the sampled rejects are given in Table 1.

### 2.2. Electrodialysis Equipment and Membranes

**Figure 2:**
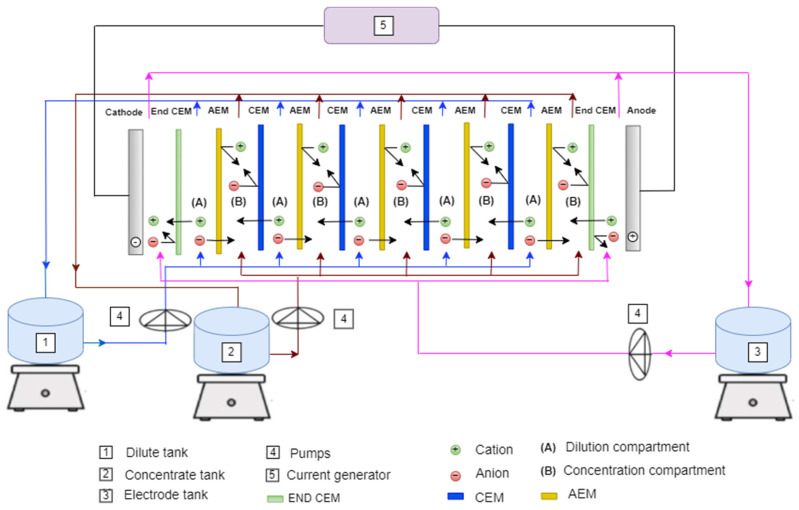
Schematic of the ED system: batch recirculation mode.

The ED configuration consists of a DC power supply, a concentrate tank, a dilute tank, a rinse electrode tank and three peristaltic pumps. (2 Masterflex l/S easy load pumps Model 7518-60), and 1 Shenchen YZ1515x Model BT 100N). Figure 2 shows a simplified schematic of ED experimental set-up operating in batch mode.

The laboratory scale ED module (64002, PCCell GmbH, Heusweiler, Germany) is composed of five cell pairs. The membranes and spacers were stacked between the two electrode end blocks. The stack consists of four (Cation Exchange Membrane CEM PC SK), two (End CEM PC-MTE), and five (Anion Exchange Membrane AEM PC SA) with an effective area of 64 cm^2^ (110 × 110 mm) for each membrane. The main characteristics of the membranes used are given in Table 2 (manufacturer data). Spacers had a thickness of 0.45 mm plastic are placed between the membranes to form the flow paths of dilute and concentrated flows. The spacers were designed to minimize boundary layer effects and were arranged in the stack so that all dilute and concentrated flows were collected separately. The ED cell consisted of two electrodes. Both electrodes are made of Pt/Ir-coated titanium

The ED stack is equipped with three separate external tanks: the first used to concentrate the solution, the second to dilute the solution, and the third to rinse the electrode solution. All three are connected to a pump for solution recirculation.

### 2.3. Experiments and Analysis Methods

#### 2.3.1. Experimental Procedure

In this study, first set of experiments were conducted with model solutions prepared with demineralized water and sodium chloride salts (NaCl). 0.05 M sodium sulfate (Na_2_SO_4_) solution was used as the rinsing solution of the electrodes circulating in the electrode compartment, to prevent the generation of chlorine or hypochlorite, which could be dangerous for the electrodes. The flow rate of the electrode rinse solution was set at 18.6 L·h^−1^ (max range for this pump) for all experiments. During all NaCl experiments, the volumes of diluted, concentrated and electrode rinse solutions were of 1 L each. The flow rate of the diluted solution varied from 20 to 55 L·h^−1^ while that of the concentrated solution was set at 20 L·h^−1^ for all experiments.

Before the onset of the desalination test, NaCl aqueous solution at the same concentration was introduced in dilute and concentrate compartments. The experiment started at the time of potential application, which varied from 6 to 12 V. The ionic conductivity was recorded every 3 min.

Then, the procedure was applied to the treatment of the real dialysis RO concentrate samples collected at with total dissolved salts ∼1.37 ± 0.06 g·L^−1^. The diluted and concentrated solutions were circulated through the ED cell until the final target conductivity (0.1 mS·cm^−1^) was reached in the diluted tank. After each experiment, ED cell was cleaned with circulation of distilled water during 30 min, followed sometimes with 0.1 M HCl solution during 15 min, in order to remove any deposits.

#### 2.3.2. Water Analysis

The pH and conductivity were measured using Consort C5010 Multi-Parameter analyzer. The other analyses were performed by the laboratory Eurofins Alpabio (Parçay Meslay, France) following the ISO standards. Briefly, the concentrations of free chlorine, total chlorine and turbidity were analyzed by spectrophotometry—NF EN ISO 7393-2. Ammonium ion concentration (NH^4+^) was analyzed by spectrophotometry method (UV/VIS)—NF ISO 15923. The concentrations for the ions calcium (Ca), magnesium (Mg), sodium (Mg), iron (Fe), arsenic (As), cadmium (Cd), and mercury (Hg) were analyzed by Inductively Coupled Plasma Mass Spectrometry: ICP/MS—NF EN ISO 17294-2. Chlorides, nitrates, and sulfates were determined by Ion Chromatography—Conductimetry—NF EN ISO 10304-1.

#### 2.3.3. Data Analysis

The ED performance under different operating conditions was evaluated based on calculations of the parameters below.

- Determination of Removal rate (R^−^%)

Removal rates of sodium, chloride, nitrate, and sulfate ions by ED technique were calculated for all experiments by the following equation [27]:

(1) $$R^{-}(\%)=100[1-(\frac{C_{f}}{C_{0}})]$$

where, C_0_ and C_f_ are the initial and final concentrations (mg·L^−1^) of the ion species (sodium, chlorine, nitrate, sulfate), respectively in the dilute compartment.

- Determination of the demineralization rate (DR %)

To study the influence of applied potential, salt concentration, and flow rate on ED efficiency, DR was calculated using the following equation: [27,28,29].

(2) $$\mathrm{DR}\;(\%)=100(1-\frac{{\mathrm{EC}}_{t}}{{\mathrm{EC}}_{0}})$$

where EC_0_ and EC_t_ are the conductivities at initial time and at time t, in the diluate compartment, respectively, expressed in mS·cm^−1^.

- Determination of the specific power consumption (SPC)

Specific power consumption (SPC) is also an important parameter of electrodialytic desalination. It can be described as the energy required to process the unit volume of solution. SPC was calculated for each experimental condition using the following equation [30]:

(3) $${\mathrm{SPC}\;(W\cdot h\cdot L}^{-1})=\frac{E\int_{0}^{t}I\left(t\right)\mathrm{dt}}{V_{d}}$$

where E (V) is the applied potential, V_d_ is volume of the diluate solution (L), and t is time.

- Determination of ion transport flux (J)

Flux values were evaluated for all experimental conditions to compare with ion transport from the dilute to the concentrate compartment. The ion flux (J) was determined using the following equation [31]:

(4) $${J\;(\mathrm{mol}\cdot \mathrm{cm}}^{-2}{\cdot s}^{-1})=(\frac{V_{d}}{A})(\frac{C_{t}{-C}_{0}}{t})$$

C_0_ and C_t_ are the concentrations at initial time and time t (mol·L^−1^)

- Determination of the productivity (W)

Productivity is a measure of the kinetic efficiency of the separation process and relates the rate of desalinated water production to the size of the system. It was calculated according to the Equation (5) below [32]:

(5) $$W\;\left(L\cdot m^{-2}\cdot h^{-1}\right)=\frac{V_{d}}{A_{\mathrm{tot}}\cdot t}$$

where A_tot_ is the area of total membrane of the stack.

### 2.4. Parametric Study and Statistical Method

In this study, a factorial design plan was proposed to investigate the performance of the ED process for the reduction of the salinity of dialysis RO concentrate. Initial salt concentration (C), dilute flow rate (Q), and applied potential (E) were chosen as relevant parameters for ED optimization. Responses were expressed in terms of demineralization rate (DR, %) and specific power consumption (SPC, Wh·L^−1^). The ranges of the operating parameters were chosen according to the capacity of our set-up and knowledge of expected conductivities [5]: 6, 9, and 12 V for voltage (E), 0.5, 1, and 1.5 g·L^−1^ for concentration (C), and 20, 37.5, and 55 L·h^−1^ for dilute flow rate (Q), respectively. The coded levels that compare the size of the coefficients on a common scale are the following: low (−1), central point (0) and high (+1) levels.

A total of 12 experiments were performed according to the complete factorial two-level—three factors (2^3^) (eight points in the factorial design and four central points to establish experimental errors). Since the interactions between these factors could be significant, a first-order linear polynomial model was postulated by the following equation:

(6) $$Y=\partial_{0}+\partial_{1}E\;+\;\partial_{2}C\;+\;\partial_{3}Q\;+\;\partial_{1.2}E.C\;+\;\partial_{1.3}E.Q\;+\;\partial_{2.3}C.Q\;+\;\partial_{1.2.3}E.C.Q$$

where Y is the response variable, ∂_0_ is the constant or intercept, ∂_1_, ∂_2_, and ∂_3_, are estimated coefficients for the linear term (also known as the slope of the line) that indicate the effect of applied potential (E), salt concentration (C), and flow rate (Q) respectively. The coefficients ∂_1.2_, ∂_1.3_, and ∂_2.3_ describe the effects of the interactions of applied salt potential concentration, applied flow potential, and concentrated salt flow concentration. The coefficient ∂_1.2.3_ implies the interaction effect between the applied salt concentration and flow potential [8,32,33].

The analysis of the experimental results was performed with statistical and graphical analysis software (Minitab version 2019). This software was used for the regression analysis of the obtained data and to estimate the coefficients of the regression equations.

## 3. Results

### 3.1. Parametric Analysis and Modeling of the ED Process with Model Solution

Before treating real RO rejects, the aim was to determine the characteristics of electrodialysis process performed by the experimental set-up with a solution modeling dialysis wastewater from RO, i.e., NaCl in demineralized water with concentrations ranging from 0.5 to 1.5 g·L^−1^, so as to cover the range of conductivity classically observed in real cases. We made this choice because the conductivity directly reflected the ionic content of the solution. Experiments were run following the design described in Materials and Methods section. All figures and tables refer to concentration variations in the dilute compartment. Thus, data for the concentrate and electrode rinse solution are not presented in this paper.

#### 3.1.1. General Trends

**Figure 3:**
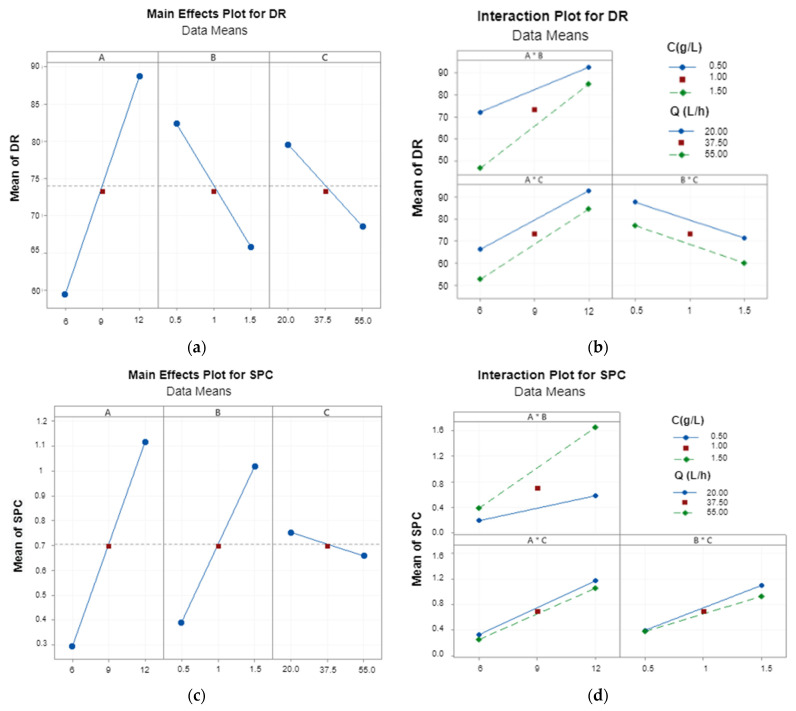
The factorial design plots of demineralization rate (DR) and specific power consumption (SPC) as a function of 3 factors A = E (V); B = C (g·L^−1^); C = Q (L·h^−1^): (**a**) main effects plots of response of DR and (**c**) SPC; (**b**) interaction plots of response of DR and (**d**) SPC.

Three factors (voltage, flow rate, initial concentration of the solution) can influence the efficiency of an electrodialysis session, estimated via the calculation of the DR and SPC parameters, as observed respectively in Figure 3a and Table S1 for DR, and in Figure 3b and Table S2 for SPC. Time was not considered here as all experiments lasted 18 min to allow comparisons.

The highest demineralization rate (DR of 94.72%) was obtained with the highest applied potential, and the lowest salt concentration and flow rate. Increasing concentration had a negative effect (negative slope) on the efficiency of desalination. An increase in concentration from 0.5 g·L^−1^ to 1.5 g·L^−1^ led to a decrease in DR of 16.6%. Thus, the separation percentage has a considerable dependence on the composition of dilute solution in this concentration range. As a general trend, an increase in the initial salt concentration from 0.5 to 1.5 g·L^−1^ resulted in a SPC increase of 63%. We noticed that an increase in flow from 20 L·h^−1^ to 55 L·h^−1^ resulted in a 11% decrease in DR. Indeed, ions did not have enough residence time to be transferred from one compartment to the other across the membrane when the tangential flux was too high. This could lead to a decrease in the total amount of ions transferred and consequently to a decrease in the separation percentage. Similar results and interpretations have been demonstrated by other groups [34,35,36].

SPC values ranged from 0.18 to 1.76 Wh·L^−1^. The lowest value of SPC was, as expected, obtained with the lowest applied potential and salt concentration and the highest flow rate. The increase in applied potential and salt concentration resulted in an increase in SPC, while flow rate had a negligible effect: a slight decrease in SPC was observed when the flow rate varied from low to high value. These results were consistent with the findings with other applications of ED, such as removal of calcium and magnesium hardness from water [37], demineralization for solutions of fish sauce [38], or lithium recovery from salt lake brines [39].

#### 3.1.2. Main Effects and Interactions between Parameters

**Figure 4:**
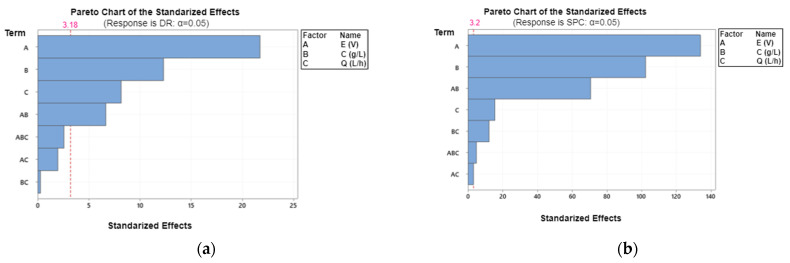
(**a**) Pareto chart for standardized effects for DR and; (**b**) SPC.

Data analysis was performed using Minitab statistical software to investigate the main effects of the factors, interactions, coefficients and of the various statistical parameters of the fitted models. The Pareto plots (Figure 4) represent the absolute values of the effects of main factors and the effects of interactions factors.

Absolute effect sizes were plotted in descending order. The significance of the individual effect of each of the variables tested at the 0.05 level was designated using a reference line on both graphs so that effects above the reference line are statistically significant at the 95% confidence level [40].

The effects of voltage, concentration, flow rate, and the interaction between voltage and concentration are the most significant for the DR (*p* < 0.05). For the SPC, it can be observed that the effects of all parameters were significant.

Based on the data presented in Figure 4 and Tables S1 and S2 in Supplementary Data, the final regression Equations (empirical models) (7) and (8) of DR and SPC in terms of coded parameters that represent the best description after the elimination of insignificant parameters (*p* > 0.05) could be determined as follows:

DR = 74.022 + 14.648A − 8.285B − 5.493C + 4.475A × B(7)

SPC = 0.70500 + 0.41250A + 0. 31500B − 0.04750C + 0.21750A × B − 0.01000A × C − 0.03750B × C − 0.01500A × B × C(8)

The estimated model for both DR and SPC had a satisfactory R^2^ greater than 99%. In the case of DR, the fit was very good (R^2^ = 99.75%) and only 0.25% of total variance was not explained by the model. The SPC (R^2^ = 99.99%) had a high value and only 0.01% of a total variance was not explained by the model.

According to the Pareto plot, voltage was the most important parameter affecting desalination efficiency and energy consumption. Indeed, an increase in the voltage value from 6 to 12 V increased the DR by 30–40%. For SPC, an increase in the voltage value increased the SPC.

The graphs of interaction effects are shown in Figure 3b,d. The non-parallel lines in this figure indicate an interaction between the two factors [41]. For DR, the results show a positive interaction between voltage and concentration (A × B), and between voltage and flow rate (A × C) but none between concentration and flow rate (B × C). For SPC, the results show a positive interaction between voltage and concentration (A × B) and no interaction between voltage and flow rate (A × C). However, there is some negative interaction between concentration and flow rate (B × C).

#### 3.1.3. Model Validation

Based on the factorial design analysis, Minitab software proposed a regression equation to predict DR and SPC for any operating conditions chosen within the min–max range:

DR = 115.2 *−* 1.52E − 64.8C *−* 1.111Q + 5.45E *×* C + 0.0911E *×* Q + 0.570C *×* Q *−* 0.0659E *×* C *×* Q (9)

SPC = 0.2121 *−* 0.02179E *−* 0.7071C *−* 0.00186Q + 0.16643E *×* C + 0.000381E *×* Q + 0.00086C *×* Q *−* 0.000571E *×* C *×* Q (10)

**Figure 5:**
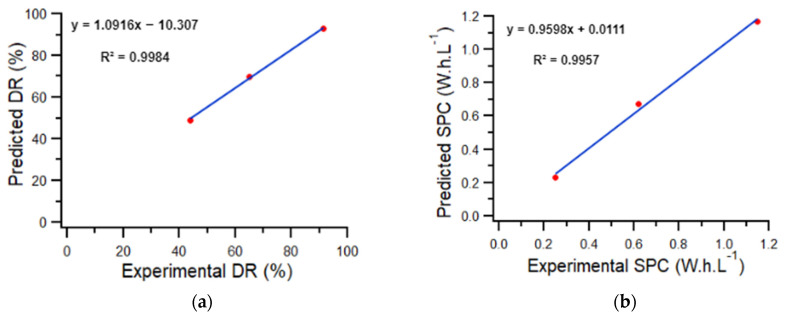
(**a**) Predicted values of the responses DR (%); (**b**) SPC (Wh·L^−1^) versus experimental values.

To validate the model, we compared its estimations with experimental responses obtained from the batch tests with three different random operating conditions (12 V, 20 L·h^−1^, 1 g·L^−1^), (9 V, 50.35 L·h^−1^, 1 g·L^−1^), and (6 V, 63.96 L·h^−1^, 1 g·L^−1^), respectively. The experimental and predicted responses of DR (%) and SPC (Wh·L^−1^) are presented in Figure 5.

The graphs show a good agreement between the experimental and the predicted values. The model shows a first order linear regression with a high coefficient of determination, R^2^ = 0.996 of DR and SPC, validating the model.

### 3.2. Application to RO Concentrate in Dialysis Unit

The operating conditions were chosen from the previous parametric study to get the highest demineralization rate with a moderate energy consumption. A compromise had thus to be found. Considering that the duration of the treatment was not a limiting factor for this application in batch mode and according to the volumes to proceed, we proposed for the further application to real RO concentrate to operate at a moderate flow rate (20 L·h^−1^), with a high voltage of 12 V applied to the ED cells.

#### 3.2.1. Comparison between Real and Model Solutions

**Figure 6:**
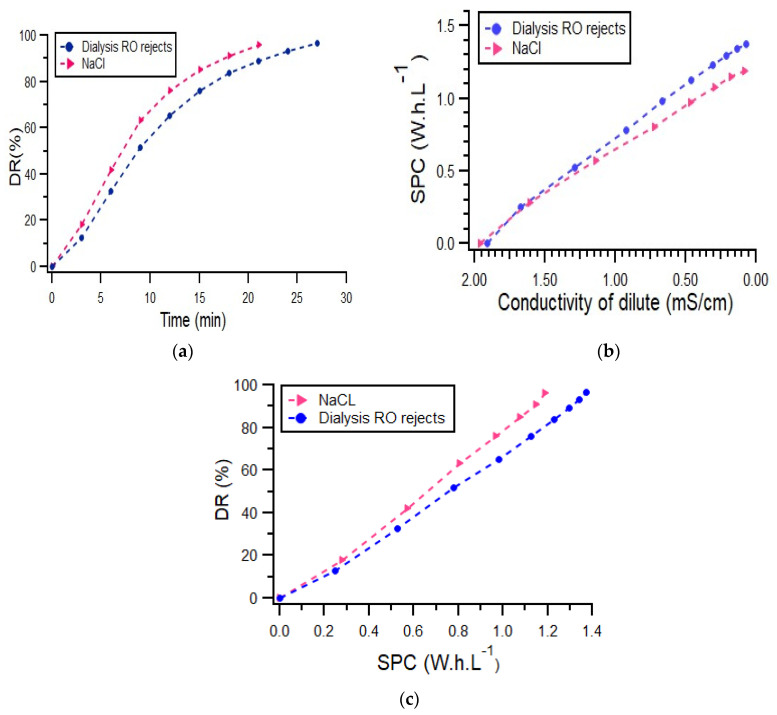
(**a**) DR (%) time course during processing of 1 L of NaCl and dialysis RO rejects; (**b**) SPC (Wh·L^−1^) consumed for the treatment 1 L of NaCl and dialysis RO rejects as a function of the conductivity (mS·cm^−1^); (**c**) Relationship between DR and SPC for the same experiments.

In a first set of experiments, we reproduced the experiments previously performed (Figure 6), with three batches of RO rejects. The mean conductivity of 1.96 mS·cm^−1^ corresponded to a NaCl concentration of 1.37 g·L^−1^, therefore in the upper range of the concentrations tested in 3.1. The same trends as those observed before are reproduced here, showing that the RO rejects solution behaved quite similarly to the NaCl one. Of note, a slight shift on the curves was observed for DR or SPC. Under these conditions, the model predicted a DR of 91.5% for a target final conductivity of 0.1 mS·cm^−1^, underestimating the values obtained with the real solution. Indeed, RO rejects are rich in other ions than Na^+^ and Cl^−^, which have also to be transferred across the ED membrane. For the same reason, SPC for RO reject water (1.37 Wh·L^−1^) was slightly higher than that of NaCl (1.19 Wh·L^−1^). In this case, the mathematical model overestimated the energy consumption with a SPC of 1.56 Wh·L^−1^. We also drew the relationship between DR and SPC, which showed that the relationship was rather linear: an increase of DR led to an increase of the duration of treatment and thus in energy consumption. As the mathematical model developed with the NaCl solution overestimated the performances, it would be necessary to adapt it to better mimic the desalination of a real RO concentrate, for further scaling-up approaches.

#### 3.2.2. Physico-Chemical Characterization of the Diluate and Species’ Mass Transfer

**Figure 7:**
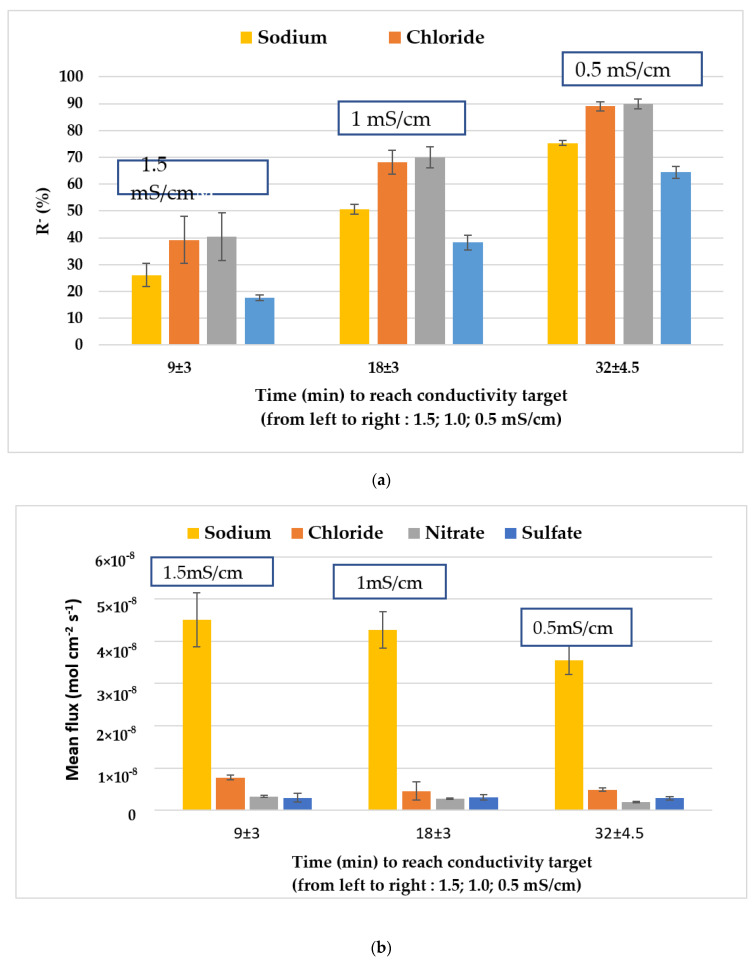
(**a**) Mean removal rate R^−^ (%) calculated rate for Na^+^, Cl^−^, NO_3_^−^, and SO_4_^2−^ for three experiments performed on dialysis RO rejects as a function of the mean time of treatment to reach each conductivity target; (**b**) variation of their fluxes as a function of time of process.

We then focused on the content of the RO concentrate and its evolution during electrodialysis treatment. Therefore, experiments were designed to achieve several target conductivities (i.e., 1.5, 1.0, 0.5, 0.1 mS·cm^−1^) and the contents of the diluate for each target were measured (Table 3), leading to the calculation of specific removal rates (Figure 7). For the foreseen re-use, we present hereafter only the results leading to a minimum conductivity of 0.5 mS·cm^−1^. For these experiments, three different batches of RO concentrates were collected at different days to account for the variability of content. Of note, the difference in treatment time to reach 0.5 mS·cm^−1^, i.e., 27 min, 33 min, and 36 min for batches 1, 2, and 3 respectively, was due to the difference in the initial conductivity of RO concentrate samples (1.87, 1.98, and 2.04 mS·cm^−1^ respectively).

For sodium and sulfate, the most abundant species, the average removal rate (%) linearly increased with the average time until conductivity reached 0.5 mS·cm^−1^ and then flattened (data not shown). The profile of the other anions tended to flatten more rapidly, because their initial concentration was much lower than that of sodium. In general, all the ions were removed by more than 90% within an average time of 66 min (i.e., for a DR of 95%).

Regarding the fluxes, the sodium flux is much higher than that of monovalent anions Cl^−^ and NO_3_^−^ which were removed from the dilute solution with about the same rate and faster than SO_4_^2−^ (Figure 7b) This is due to its relatively high abundance until the end of the experiment. The decrease in average flux as a function of average time is explained by the decrease in current density averages (data not shown). The rate of ion transport across membranes is determined by both the concentration and mobility of ions in the membranes. The rate of transport is related to the valence and size of the ions: ions with higher valence, or larger Stokes radii move slowly compared to ions with lower valence, and smaller Stokes radii [42]. The ionic Stokes radii of sodium, chloride, nitrate and sulfate are respectively 1.84 Å, 1.21 Å, 1.29 Å, and 2.30 Å [43]. Sulfate ions have a larger Stokes radius than chloride and nitrate ions. Therefore, they experienced a higher resistance when migrating through the membrane, which explains their low flux and low removal rate as well (b) [44]. Sulfate is removed with a lower rate, but this rate increased as the concentration of competing ions decrease.

As sodium is the major ion in the RO rejects, and all ions were correctly removed, the follow-up of conductivity can be considered as sufficient to determine ions’ removal in this fluid.

## 4. Discussion

Several solutions can be proposed for the re-use of the treated RO concentrates. The associated standards or requirements collected from several institutions are provided in Table 4 and for specific content in Supplementary Data (Table S3). It should be noticed that, depending on the application, decreasing the conductivity to the limit given by the standards might not be enough to fulfill all the requirements (for instance, pH or a specific ion concentration might not be correct). Therefore, based on the analysis performed in 3.2, we propose in this table a target as “re-use conductivity” that might be different from the data found in standard.

Assuming that the initial conductivity is about 2 mS·cm^−1^, DR from 50% to 75% were necessary to fulfill the requirement for re-use. This can be achieved in any condition with a moderate energy consumption (Table 4).

Water for irrigation presented the most drastic requirements, with a target conductivity between 0.3 and 0.7 mS·cm^−1^. This application might thus not be the best option for re-use, although concentrations in chloride, nitrate, and sulfate achieved with this conductivity were much below the threshold values fixed by WHO. These data confirmed that ED is probably the best choice for treating this reject, compared to reverse osmosis process which is more energy demanding for the same DR, and thus less cost- effective. In a very recent paper, Patel et al. (2021), showed that ED exceled at low salinities (<3 g·L^−1^) [48]. The cost of desalination is proportional to the amount of salt removed [47,48,49]. Based on its current application, it can treat reverse osmosis effluent from dialysis centers where salinities remained below 2.0 g·L^−1^ at all sites.

**Figure 8:**
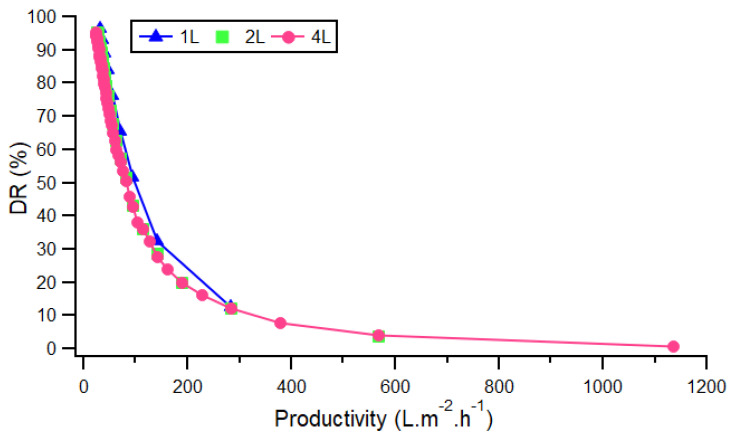
Relationship between demineralization rate and productivity with different volumes of dialysis RO treated.

Finally, in a scaling up approach, another important issue was to define a range of productivity for the operation. The productivity gives information about the kinetics efficacy of the separation process, as defined in Equation (5). Figure 8 shows that the productivity was independent from the diluate volumes treated for the same concentrate volume (different recovery rate), and varied mainly with the target DR. The productivities obtained for our application were in the same range or even higher than the few ones found in the literature [50,51]. Although it was difficult to compare data since the initial concentrations of brackish water were not similar to our RO concentrates, we considered that these good results came from the optimization of process parameters we proposed in the first part of the study. In addition, in a circular economy perspective, recent work regarding the re-use of old reverse osmosis membranes in a ED unit could be a way to alleviate the environmental burden of the water treatment system in hemodialysis [52].

The knowledge of productivity will help in the choice of the membrane surface area, while that of SPC will give some indication regarding energy consumption. As an example, the dialysis center at Polyclinique Saint-Côme rejects 9 m^3^ of water issued from the RO loop per day. To re-use this volume for manual sterilization in the clinics, a 3 m^2^-membrane area ED system would be necessary. Operating with a high recovery rate (from 80 to 88%) produces concentrates of electrodialysis with conductivities from 6 to 10 mS·cm^−1^. These values are compatible with a reject in the sewage system. The corresponding energy consumption would be about 6.5 kWh, for the ED equipment only (without the pumps). In a green perspective, energy supply should be provided by solar energy, reducing the environmental impact of the additional energy requested. According to the above energy demand, 90 m^2^ of photovoltaic panels would be able to supply the ED modules. Such plants were already proposed and deployed for desalination process of brackish water [53], proving their efficacy with moderate salt concentration, as it is the case for the dialysis RO concentrate.

## 5. Conclusions

The large volumes of water rejected by the practice of hemodialysis make it relevant to study their potential for reuse. RO concentrates contain middle concentrations of sodium, sulfate, nitrate, and chloride ions. At laboratory scale, ED has been shown to be an effective method from removing excess ions from RO dialysis rejects meeting requirements for several re-uses such as in rehabilitation pools or pre-sterilization cleaning. The next step will be the built-up of a pilot unit to demonstrate the feasibility of its use at larger scale.

## Figures and Tables

**Table 1:** Physical and chemical parameters measured in dialysis RO rejects from Polyclinic Saint Côme (N = 3).

Parameter	Dialysis RO Loop Water Reject
	Mean (±SD)
Conductivity (μS·cm^−1^)	1960 ± 0.086
pH	8.12 ± 0.22
Calcium, mmol·L^−1^	0.025 ± 3.06 × 10^−3^
Chloride, mmol·L^−1^	2.183 ± 0.115
Total Hardness, °F	0.32 ± 0.04
Magnesium, mmol·L^−1^	0.005 ± 9.12 × 10^−4^
Sodium, mmol·L^−1^	19.164 ± 0.7844
Sulfates, mmol·L^−1^	1.77 ± 0.104
Turbidity, NFU	0.10 ± 0,00
Ammonium, mmol·L^−1^	<0.0027 ± 0.00
Nitrates, mmol·L^−1^	0.0715 ± 0.0042
Free Chlorine, mmol·L^−1^	<0.00006 ± 0.00
Total Chlorine, mmol·L^−1^	0.00007 ± 2 × 10^−5^
Iron, μmol·L^−1^	0.07 ± 0.01
Arsenic, μmol·L^−1^	0.00253 ± 2.02 × 10^−4^
Cadmium, μmol·L^−1^	<0.000088 ± 0.00
Mercury, μmol·L^−1^	<0.00005 ± 0.00

**Table 2:** Parameters of the experimental membranes according to the manufacturer.

Membrane Type	Membrane Characteristic	Resistance/ Ω·cm^2^	Water Content (wt%)	Thickness (μm)	Ion Exchange Capacity Strong Basic (mequiv·g^−1^)	Chemical Stability (pH)	Permselectivity
PC-SK	Strongly acidic (Sulfonic acid)	~2.5	~9	100–120	c.a. 1.2	0–11	>0.95
PC-SA	Strongly alkaline (Ammonium)	~1.8	~14	100–110	3	0–9	>0.95
PC -MTE	Strongly acidic (Sulfonic acid)	~4.5	-	220	1.8	1–13	>0.94

**Table 3:** Physico-chemical characterization of dialysis RO concentrates to reach conductivity targets: 1.5, 1.0, and 0.5 mS·cm^−1^, respectively.

Parameter	Unit	Dialysis Loop Water RO before ED	Target1.5 mS·cm^−1^	ED Target1 mS·cm^−1^	ED Target0.5 mS·cm^−1^
		Mean (±SD)	Mean (±SD)	Mean (±SD)	Mean (±SD)
Conductivity	(mS·cm^−1^)	1.96 ± 0.092	1.50 ± 0.030	1.04 ± 0.060	0.54 ± 0.0081
pH		8.12 ± 0.22	7.79 ± 0.34	7.72 ± 0.37	7.36 ± 0.39
Calcium	mg·L^−1^	1.06 ± 0.13	0.77 ± 0.12	0.49 ± 0.06	0.26 ± 0.02
Chloride	mg·L^−1^	77.67 ± 4.04	50.33 ± 9.71	24.67 ± 2.08	8.50 ± 0.87
Total Hardness	° f	0.32 ± 0.04	0.23 ± 0.04	0.15 ± 0.02	0.07 ± 0.00
Magnesium	mg·L^−1^	0.12 ± 0.2	0.09 ± 0.03	0.05 ± 0.01	0.02 ± 0.01
Sodium	mg·L^−1^	440.67 ± 17.93	325.00 ± 10.58	217.00 ± 1.73	108.67 ± 1.15
Sulfates	mg·L^−1^	170.00 ± 10.00	140.00 ± 10.00	105.00 ± 8.66	60.67 ± 6.81
Turbidity	NFU	0.10 ± 000	0.23 ± 0.06	0.20 ± 0.10	0.13 ± 0.06
Ammonium	mg·L^−1^	<0.05 ± 0.00	<0.05 ± 0.00	<0.05 ± 0.00	<0.05 ± 0.00
Nitrates	mg·L^−1^	55.67 ± 3.21	33.00 ± 3.00	16.67 ± 1.15	5.60 ± 0.70
Free Chlorine	mg·L^−1^	<0.02 ± 0.00	<0.02 ± 0.00	<0.02 ± 0.00	<0.02 ± 0.00
Total Chlorine	mg·L^−1^	0.03 ± 0.00	0.03 ± 0.00	<0.02 ± 0.00	0.03 ± 0.00
Iron	μg·L^−1^	3.67 ± 0.58	3.50 ± 2.12	1.33 ± 0.58	1.67 ± 0.58
Arsenic	μg·L^−1^	0.19 ± 0.02	0.21 ± 0.11	0.16 ± 0.06	0.09 ± 0.02
Cadmium	μg·L^−1^	<0.01 ± 0.00	<0.01 ± 0.00	<0.01 ± 0.00	<0.01 ± 0.00
Mercury	μg·L^−1^	<0.01 ± 0.00	<0.01 ± 0.00	<0.01 ± 0.00	<0.01 ± 0.00

**Table 4:** Relevant performance parameters to achieve water quality standards for each reuse of dialysis RO concentrates.

Potential Use of Treated RO Effluents	Re-Use Conductivity (mS·cm^−1^)	DR (%)	SPC (kW·h·m^−3^)	W (L·m^−2^·h^−1^)
Rehabilitation pool [45]	1.05	45	0.69	107
Sterilization: ManualWashing [46]	1	48	0.72	102
Sterilization: Machine wash and vacuum pump [46]	0.65	66	0.98	70
Irrigation of green areas [47]	0.5	74	1.09	59

## Data Availability

Data are available upon request and stored in-house.

## Supplementary Materials

The following are available online at https://www.mdpi.com/article/10.3390/membranes12010045/s1, Table S1: Estimated effects and coefficients for DR; Table S2: Estimated effects and coefficients for SPC; Table S3: Physicochemical quality of water for each reuse.
