# Supplementary material for: Electrodialysis Can Lower the Environmental Impact of Hemodialysis
Source: Membranes (Basel). 2021 Dec 29;12(1):45. doi: 10.3390/membranes12010045 (PMC8779760; doi:10.3390/membranes12010045)
Supplement: Supplementary file 1 [file membranes-12-00045-s001.zip › membranes-1481312-supplementary.pdf]

Supplementary Materials

Electrodialysis Can Lower the Environmental Impact of Hemodialysis

Ahmed Abarkan [1,2], Nabil Grimi [3], Hubert Métayer [4], Tarik Sqalli Houssaïni [2,5] and Cecile Legallais [1,*]

[1] Biomechanics & Bioengineering Laboratory, CNRS, Université de Technologie de Compiegne, 60203 Compiègne, France; ahmed.abarkan@utc.fr

[2] Laboratory of Epidemiology and Research in Health Sciences (ERESS), Faculty of Medicine and Pharmacy, Sidi Mohammed Ben Abdellah University, Fez 30050, Morocco; tarik.sqalli@usmba.ac.ma

[3] Université de Technologie de Compiègne, ESCOM, TIMR (Transformations Intégrées de la Matière Renouvelable), Centre de Recherche Royallieu–CS 60319, 60203 Compiègne, France; nabil.grimi@utc.fr

[4] Hemodialysis Department of the Polyclinic Saint-Côme and Dialoise Self-Dialysis Center, 60200 Compiègne, France ; hubert.metayer@stcome.com

[5] Department of Nephrology, University Hospital Hassan II, Fez 30050, Morocco

* Correspondence: cecile.legallais@utc.fr

Table S1: Estimated effects and coefficients for DR.

(coded units)

Term	Effect	Coefficient	Value of T	Value of p
Costant		74.022	109.88	0.000
A	29.295	14.648	21.74	0.000
B	-16.570	-8.285	-12.30	0.001
C	-10.985	-5.493	-8.15	0.004
A*B	8.950	4.475	6.64	0.007
A*C	2.645	1.323	1.96	0.144
B*C	-0.400	-0.200	-0.30	0.786
A*B*C	-3.460	-1.730	-2.57	0.083

Standard error coefficient for all cases = 0.674 $R^2 = 0.9960$

Table S2: Estimated effects and coefficients for SPC.

(coded units)

Term	Effect	Coefficient	Value of T	Value of p
Constant		0.70500	228.73	0.000
A	0.82500	0.41250	133.83	0.000
B	0.63000	0.31500	102.20	0.000
C	-0.09500	-0.04750	-15.41	0.001
A*B	0.43500	0.21750	70.57	0.000
A*C	-0.02000	-0.01000	-3.24	0.048
B*C	-0.07500	-0.03750	-12.17	0.001
A*B*C	-0.03000	-0.01500	-4.87	0.017

Standard error coefficient for all cases = 0.674 $R^2 = 0.9960$

Table S3: Physicochemical quality of water for each reuse.

Physico-chemical parameters	Rehabilitation pool [45]	Sterilization: Manual Washing [46]	Sterilization: Machine wash and vaccum pump [46]	Irrigation [47]
Conductivity µS.cm^{-1}		200-1100	200-1100	300-700
pH	6.7 - 7.7	6.5 – 9	5-7.5	6.5-8.4
Ammonium mg.L^{-1}		≤ 0.1		
Sodium mg.L^{-1}		≤ 200		
Free chlorine mg.L^{-1}	0.4 - 1.4	≤ 0.1	≤ 0.1	
Hardness (TH) F°		15 – 30	< 0.5	
Chlorides mg.L^{-1}	> 250	≤ 250	≤ 250	142-355
Nitrate mg.L^{-1}				30
Sulfate mg.L^{-1}				250
Inorganic silicate (SiO2) mg.L^{-1}			10-20	
Total iron µg .L^{-1}		≤ 0.2	≤ 0.2	
Cadmium µg .L^{-1}		≤ 5	≤ 5	
Lead µg.L^{-1}		≤ 10	≤ 10	
Temperature °C	> 36			
Transparency	good			
